# Permitted water pollution discharges and population cancer and non-cancer mortality: toxicity weights and upstream discharge effects in US rural-urban areas

**DOI:** 10.1186/1476-072X-11-9

**Published:** 2012-04-02

**Authors:** Michael Hendryx, Jamison Conley, Evan Fedorko, Juhua Luo, Matthew Armistead

**Affiliations:** 1 West Virginia Rural Health Research Center, West Virginia University, Morgantown, USA; 2 Department of Community Medicine, West Virginia University, Morgantown, USA; 3 Department of Geology and Geography, West Virginia University, Morgantown, USA; 4 Department of Community Medicine, West Virginia University, PO Box 9190, Morgantown, WV 26505, USA

**Keywords:** Age-adjusted mortality, Spatial analysis, Water pollution, Cancer, Kidney disease, Rural-urban differences

## Abstract

**Background:**

The study conducts statistical and spatial analyses to investigate amounts and types of permitted surface water pollution discharges in relation to population mortality rates for cancer and non-cancer causes nationwide and by urban-rural setting. Data from the Environmental Protection Agency's (EPA) Discharge Monitoring Report (DMR) were used to measure the location, type, and quantity of a selected set of 38 discharge chemicals for 10,395 facilities across the contiguous US. Exposures were refined by weighting amounts of chemical discharges by their estimated toxicity to human health, and by estimating the discharges that occur not only in a local county, but area-weighted discharges occurring upstream in the same watershed. Centers for Disease Control and Prevention (CDC) mortality files were used to measure age-adjusted population mortality rates for cancer, kidney disease, and total non-cancer causes. Analysis included multiple linear regressions to adjust for population health risk covariates. Spatial analyses were conducted by applying geographically weighted regression to examine the geographic relationships between releases and mortality.

**Results:**

Greater non-carcinogenic chemical discharge quantities were associated with significantly higher non-cancer mortality rates, regardless of toxicity weighting or upstream discharge weighting. Cancer mortality was higher in association with carcinogenic discharges only after applying toxicity weights. Kidney disease mortality was related to higher non-carcinogenic discharges only when both applying toxicity weights and including upstream discharges. Effects for kidney mortality and total non-cancer mortality were stronger in rural areas than urban areas. Spatial results show correlations between non-carcinogenic discharges and cancer mortality for much of the contiguous United States, suggesting that chemicals not currently recognized as carcinogens may contribute to cancer mortality risk. The geographically weighted regression results suggest spatial variability in effects, and also indicate that some rural communities may be impacted by upstream urban discharges.

**Conclusions:**

There is evidence that permitted surface water chemical discharges are related to population mortality. Toxicity weights and upstream discharges are important for understanding some mortality effects. Chemicals not currently recognized as carcinogens may nevertheless play a role in contributing to cancer mortality risk. Spatial models allow for the examination of geographic variability not captured through the regression models.

## Background

A variety of water quality issues potentially impact rural and urban populations. Previous research identified 82,498 EPA-permitted water point pollution discharge sources in the US, of which 41% were located in rural areas of the country [1]. Discharge of pollutants into surface water also has potential downstream impacts that may cross between urban and rural settings [2,3]. Drinking water containing carcinogens such as arsenic or cadmium has been linked to various cancers and other diseases [4,5].

There are many industrial water pollutants that may potentially impact human health. Exposure routes include both inhalation and ingestion of drinking water. Contaminated ground water in areas with hazardous waste sites has been shown to correlate with higher population cancer mortality rates and other human disease rates [6,7]. Epidemiological research to investigate whether and how health may be influenced by industrial water pollutants is limited [4,8], and research on the population health risks from the *permitted *surface water pollution discharge database represented in this study has apparently not been undertaken. Surface and ground water are interrelated and surface pollution can impair ground water [9].

In this study, we test the hypothesis that greater amounts of permitted toxic chemical pollutants in surface water will be associated with poorer population health. We are also interested in testing whether there is evidence for pollution discharges affecting population health downstream from its source, and whether these associations may be present differently between rural and urban environments. This is an exploratory study intended to establish whether associations exist between discharges and health outcomes; if such evidence is found, more specific hypotheses may be generated regarding relationships between specific chemicals and outcomes that may vary by geographic location as suggestions to encourage future research.

## Results and discussion

### Non-spatial

Table 1 presents summary statistics of the variables used in the study. The study N = 3,083 represents US counties with complete data on measures of interest. Mortality rates for kidney disease were available for 2,400 counties due to CDC suppression of values because of small numbers of cases.

**Table 1:** Descriptive statistics of study variables

Dependent Variable	Mean	Standard Deviation
Total age-adjusted mortality rate per 100,000 for non- cancer causes	658.3	112.2

Age-adjusted all-cancer mortality rate per 100,000	197.6	29.4

Age-adjusted kidney disease mortality rate per 100,000	17.6	7.3

Independent Variables		

Log of non-weighted, onsite non-carcinogenic discharges	2.59	2.82

Log of non-weighted, onsite carcinogenic discharges	0.22	0.89

Log of toxicity-weighted, onsite non-carcinogenic discharges	5.69	4.82

Log of toxicity-weighted, onsite carcinogenic discharges	2.36	5.15

Log of toxicity-weighted, local and upstream non- carcinogenic discharges	1.29	1.90

Log of toxicity-weighted, local and upstream carcinogenic discharges	1.58	2.80

Covariates		

Percent adults aged 25+ with college or more education	16.5	7.8

Adult smoking rate	21.8	4.3

Adult obesity rate	28.9	3.7

Primary care physicians per 1,000 population	0.4	0.3

Poverty rate	15.1	6.2

Percent African American	8.9	14.6

Percent Native American	1.6	6.4

Percent Hispanic	6.2	12.0

Percent Asian American	0.8	1.6

Percent other non-White race	2.6	4.8

Percent White	84.7	16.1

Percent metropolitan county	35.2	47.8

Percent non-metropolitan, adjacent county	46.7	49.9

Percent non-metropolitan, non-adjacent	18.1	38.5

Table 2 includes the summary of regression coefficients in the models for analysis Sets 1 through 4. For total non-cancer mortality, greater discharges of non-carcinogenic chemicals were associated with higher mortality rates for Set 1, and remained significant in Sets 2 and 3. For cancer mortality, onsite carcinogenic discharges were not associated with death rates before toxicity weighting, but were significantly associated with death rates after toxicity weighting. For kidney disease, non-carcinogenic discharges were not related to death rates in Sets 1 and 2, but when discharges were both toxicity weighted and area weighted to account for upstream discharges, higher discharge levels were significantly related to higher death rates.

**Table 2:** Multiple regression coefficients, standard errors (SE), and p-values, age-adjusted mortality rates and four discharge specifications

	Set 1: Log of onsite discharges not toxicity weighted		Set 2: Log of onsite discharges toxicity weighted		Set 3: Log of area weighted upstream discharges, toxicity weighted		Set 4: Log of area weighted upstream discharges, toxicity weighted, cross-validation	
	**Coeff. (SE)**	**P <**	**Coeff. (SE)**	**P <**	**Coeff. (SE)**	**P <**	**Coeff. (SE)**	**P <**
**All-Cancer mortality**	0.74 (.52)	0.16	0.20 (.09)	0.03	0.35 (.16)	0.03	0.98 (.24)	0.0001
**Kidney disease mortality**	-.02 (.05)	0.63	-.02 (.03)	0.40	0.25 (.06)	0.0001	0.11 (.04)	0.01
**Total non-cancer mortality**	2.94 (.48)	0.0001	1.82 (.28)	0.0001	2.30 (.69)	0.0009	0.32 (.46)	0.49

Models control for college education rates, smoking rates, adult obesity rates, supply of primary care physicians, poverty rate, percent African American, percent Native American, percent non-white Hispanic, percent Asian American, percent other non-white race (percent white serving as the referent), metropolitan county, and non-metropolitan adjacent county (non-metropolitan and non-adjacent county serving as the referent.) Model F values for all models were significant at p < .0001

Table 2 also shows the results of the cross-validation analyses as Set 4. In this analysis, area weighted and toxicity weighted discharges constitute the primary independent variable of interest. For cancer mortality, we observed an unexpected finding, namely, that non-carcinogen discharges were related to higher mortality at a more stringent p value than carcinogen discharges. For kidney disease the effect was stronger for non-carcinogen discharges as expected, but p values were significant for both discharge types. For total non-cancer mortality, only non-carcinogen discharges were related to a higher mortality rate.

Table 3 shows the results from the Set 5 analyses specific to metropolitan, and adjacent and non-adjacent non-metropolitan areas. We are particularly interested here in whether or not death rates in non-metropolitan areas may be related to discharges using the area weighted and toxicity weighted variable, reflective of upstream discharges that may affect downstream rural areas. For cancer mortality, the significant effect observed for Set 3 (Table 2) is not specific to rural-urban specification. For kidney disease and total non-cancer mortality, however, the significant effects observed for Set 3 (Table 2) are significant only in non-adjacent non-metropolitan areas. Death rates for total non-cancer and kidney disease in rural areas that are not adjacent to metropolitan areas are higher in association with greater local and upstream toxicity-weighted water pollution discharges.

**Table 3:** Multiple regression coefficients, standard errors (SE), and p-values.

	Metropolitan		Adjacent non- metropolitan		Non-adjacent non- metropolitan	
	**Coeff. (SE)**	**P <**	**Coeff. (SE)**	**P <**	**Coeff. (SE)**	**P <**
**All-Cancer mortality**	0.32 (.18)	0.08	0.38 (.28)	0.18	0.32 (.51)	0.53
**Kidney disease mortality**	0.14 (.08)	0.07	0.21 (.12)	0.08	0.55 (.18)	0.003
**Total non-cancer mortality**	1.21 (.82)	0.15	1.41 (1.20)	0.25	6.85 (2.17)	0.002

age-adjusted mortality rates and discharges by metropolitan status

Models control for college education rates, smoking rates, adult obesity rates, supply of primary care physicians, poverty rate, percent African American, percent Native American, percent non-white Hispanic, percent Asian American, and percent other non-white race (percent white serving as the referent). Model F values for all models significant at p < .0001

Finally, Table 4 shows the full results for Set 3 including all covariates. Variables such as higher smoking and obesity rates, higher poverty rates, and lower education levels were associated with higher mortality rates. Higher mortality rates were generally associated with more urban settings, and with larger percent populations of African Americans and 'other' non-white race.

**Table 4:** Multiple regression results including covariates, for age-adjusted mortality rates and area-weighted and toxicity weighted discharges

	**All-Cancer mortality**^**1**^		**Kidney disease mortality**^**2**^		**Total non- cancer mortality**^**3**^	
	**Coeff. (SE)**	**P <**	**Coeff. (SE)**	**P <**	**Coeff. (SE)**	**P <**
**Log of non-carcinogen area weighted and toxicity weighted discharges**	NA	--	0.25 (.06)	< 0.0001	2.30 (.69)	0.0009
**Log of carcinogen area weighted and toxicity weighted discharges**	0.35 (.16)	0.03	NA	--	NA	--
**Percent adults with college education**	-0.78 (.09)	< 0.0001	-0.11 (.02)	< 0.0001	-28 .5	< 0.0001
**Adult smoking rate**	1.24 (.12)	< 0.0001	0.18 (.03)	< 0.0001	3.25 (.35)	< 0.0001
**Adult obesity rate**	0.43 (.18)	0.02	0.17 (.05)	0.002	2.88 (.52)	< 0.0001
**Per capita primary care doctors**	1.83 (1.61)	0.26	-0.47 (.52)	0.37	7.03 (4.66)	0.14
**Poverty rate**	1.11 (.11)	< 0.0001	0.20 (.03)	< 0.0001	5.38 (.30)	< 0.0001
**Percent African American**	0.20 (.04)	< 0.0001	0.14 (.01)	< 0.0001	1.50 (.12)	< 0.0001
**Percent Native American**	-0.22 (.08)	0.004	0.03 (.03)	0.23	0.45 (.22)	0.04
**Percent Hispanic**	-0.91 (.09)	< 0.0001	0.03 (.03)	0.36	-2.16 (.27)	< 0.0001
**Percent Asian American**	0.65 (.34)	0.07	-0.19 (.09)	0.04	-0.07 (.99)	0.94
**Percent other race**	0.76 (.22)	0.0007	-0.17 (.07)	0.02	3.63 (.65)	< 0.0001
**Metropolitan county**	9.98 (1.39)	< 0.0001	0.02 (.40)	0.97	38.52 (4.03)	< 0.0001
**Adjacent, non- metropolitan county**	1.90 (1.22)	0.12	-0.09 (.37)	0.82	9.00 (3.52)	0.02

1. Model F = 123.1 (df = 13, 3068), p < .0001; adjusted R-square = .34

2. Model F = 97.9 (df = 13, 2384), p < .0001; adjusted R-square = .34

3. Model F = 255.6 (df = 13, 3068), p < .0001; adjusted R-square = .52

### Spatial

A test for spatial autocorrelation of the residuals from the ordinary least squares regression shows that there is significant autocorrelation among the residuals (Moran's *I *= 0.107, *p *< 0.001, inverse distance spatial weights matrix). The significance of this test suggests that either this model is missing one or more useful covariates or a spatial approach such as geographically weighted regression (GWR) may be appropriate [10]. GWR is described more fully in the methods section.

**Figure 1:**
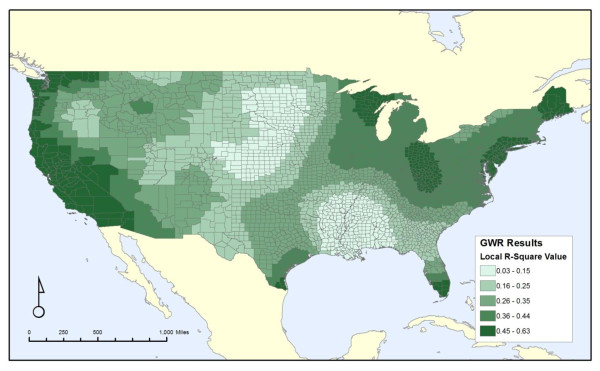
**Local R-Square values for geographic-weighted regression results for cancer mortality and area weighted and toxicity-weighted release**.

The first GWR analysis (GWR set A) examines area-weighted and toxicity-weighted carcinogenic discharges, which is equivalent to the non-spatial carcinogen analysis of Set 3, in relation to cancer mortality. The local R^2 ^map (Figure 1) shows a large region of very low values along the lower Mississippi River valley and in much of the Great Plains, while higher values are found in parts of the Midwest and along both the Pacific and Atlantic coasts.

**Figure 2:**
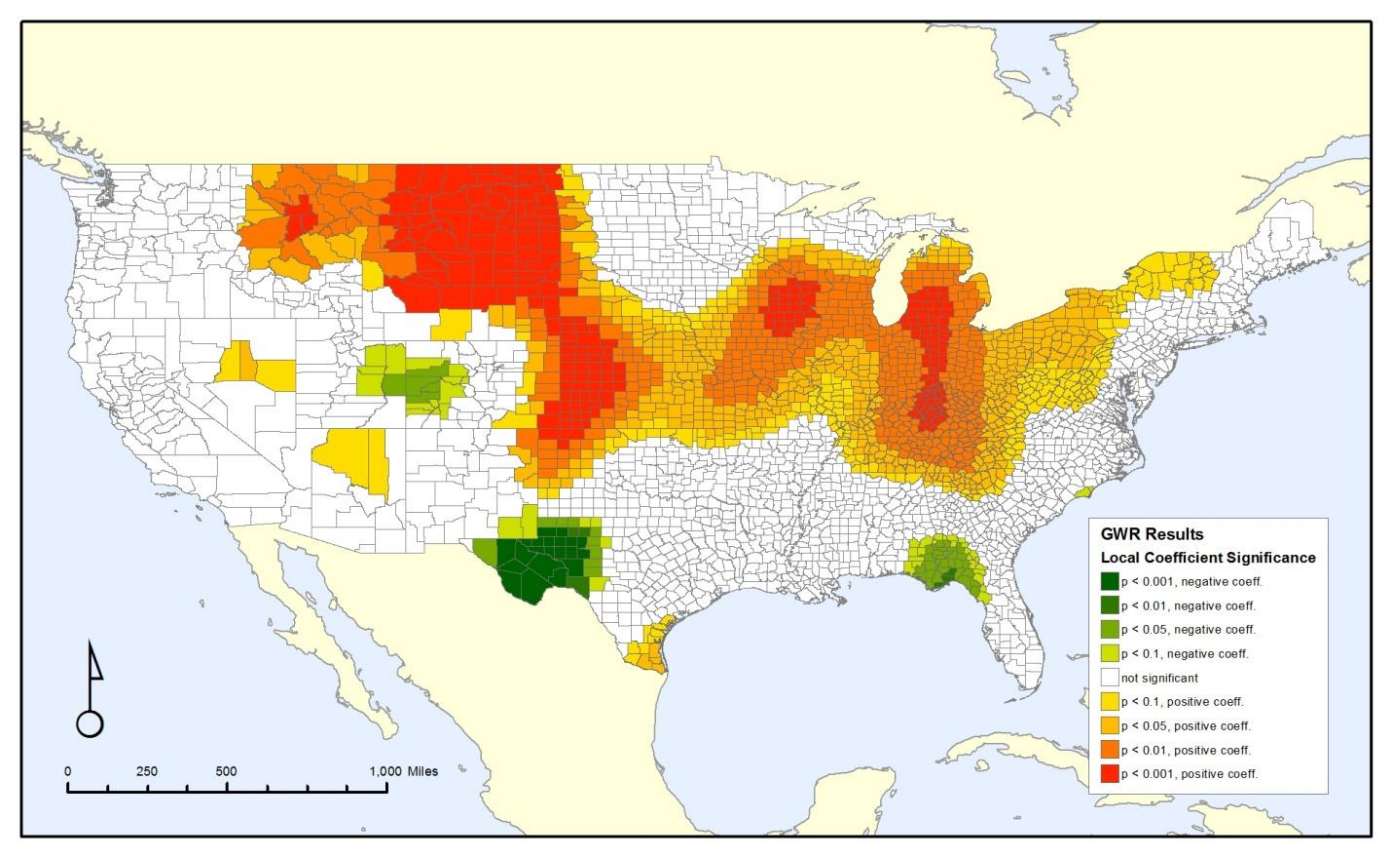
**Local geographic-weighted regression coefficients for all-cancer mortality and area-weighted, toxicity-weighted carcinogenic discharges**.

Figure 2 displays a map of the significance of the local regression coefficient of the release variable, highlighting which parts of the country have the strongest relationship between cancer mortality and the area-weighted, toxicity-weighted measure of carcinogenic discharges. There is a broad area of significantly positive coefficients stretching from the northern Rocky Mountains to the Ohio and Tennessee River Valleys. Meanwhile, there are only a few small pockets of negative coefficients, with the most significant of those being in western Texas. Results of all seven analyses are not shown to conserve space, and are available from the authors on request.

**Figure 3:**
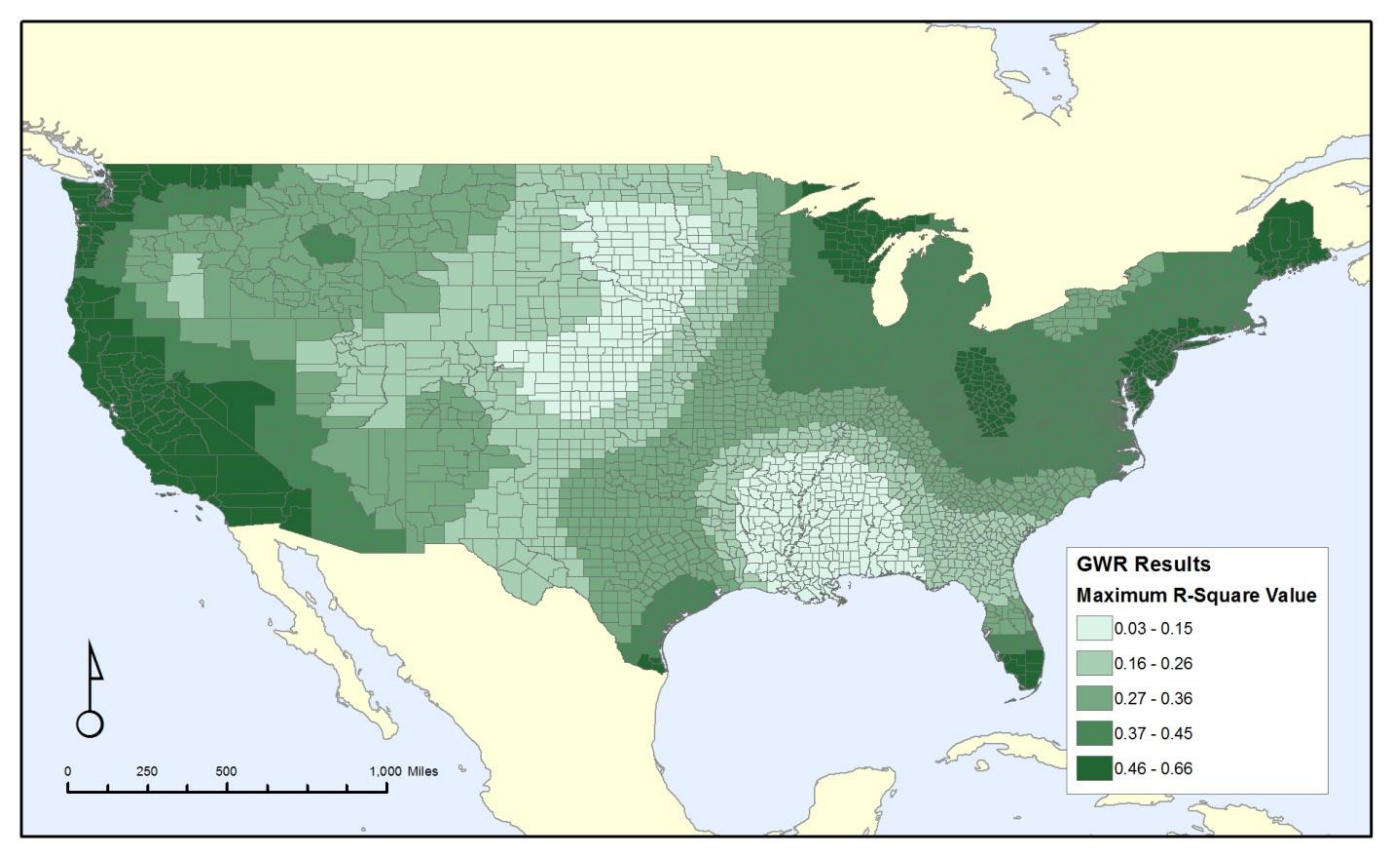
**Maximum local R^2 ^values for all-cancer mortality across all release variables**.

Figure 3 shows the maximum local R^2 ^from all seven GWR analyses. The broad pattern introduced in Figure 1 of low values along the lower Mississippi River and in the Great Plains persists across all GWR results, along with higher values along the Pacific coast and in parts of the Midwest and Northeast. There is a wide range of local R^2 ^values from less than 0.03 to greater than 0.65, demonstrating that while the discharges and covariates may correlate well with cancer mortality in some regions of the country, they do not provide a strong correlation nationwide. This also demonstrates that the non-spatial analyses are masking substantial regional variation in the correlations between these discharges and health outcomes.

**Figure 4:**
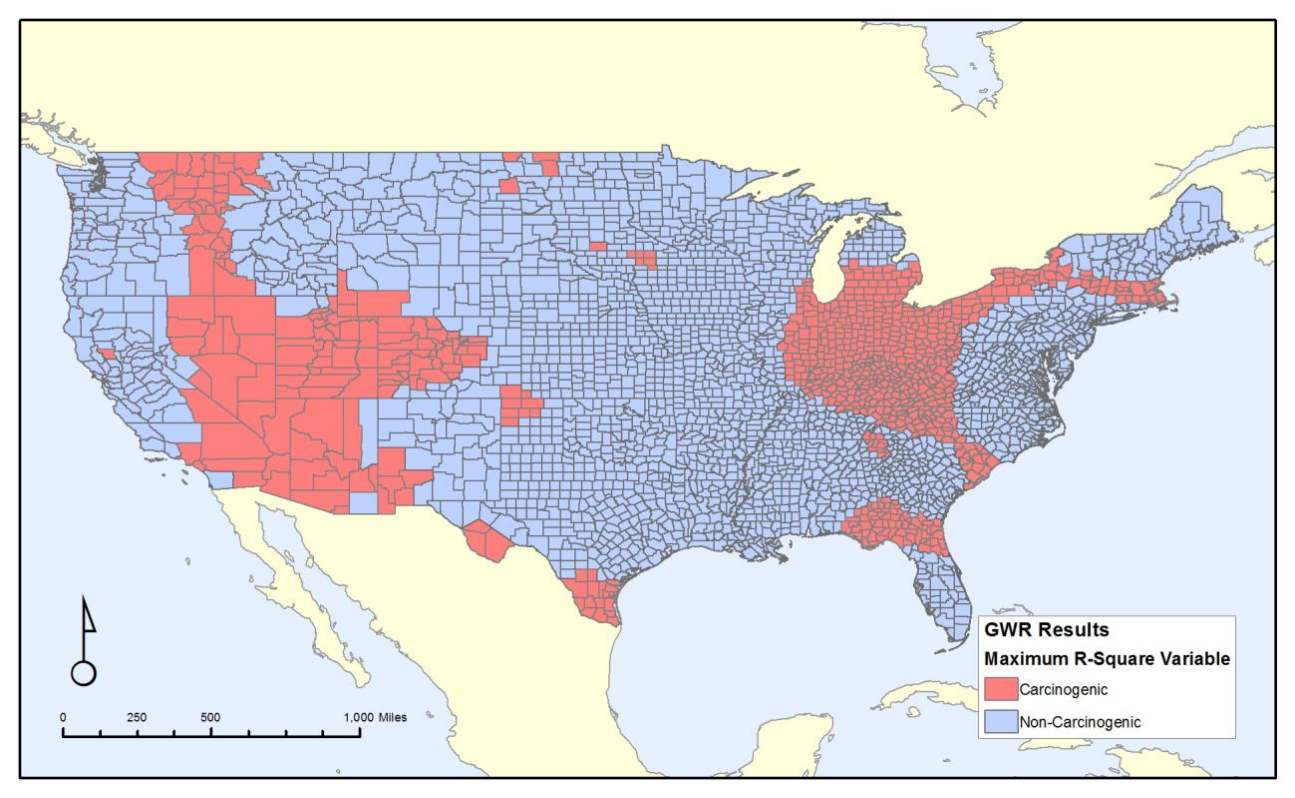
**Regions where carcinogens versus non-carcinogens had the greatest local correlation with all-cancer mortality**.

**Figure 5:**
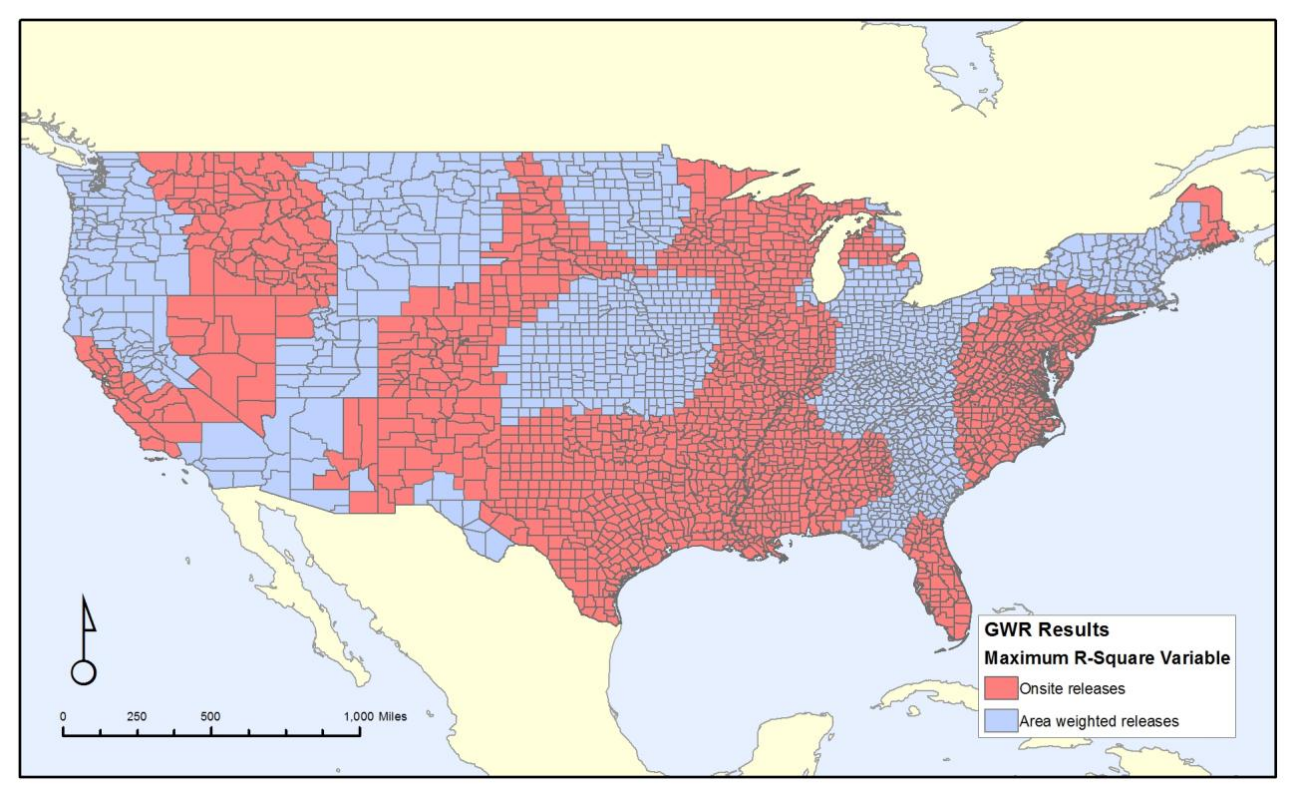
**Regions where onsite releases in the county versus an area-weighted average of all upstream releases had the greatest local correlation with all-cancer mortality**.

**Figure 6:**
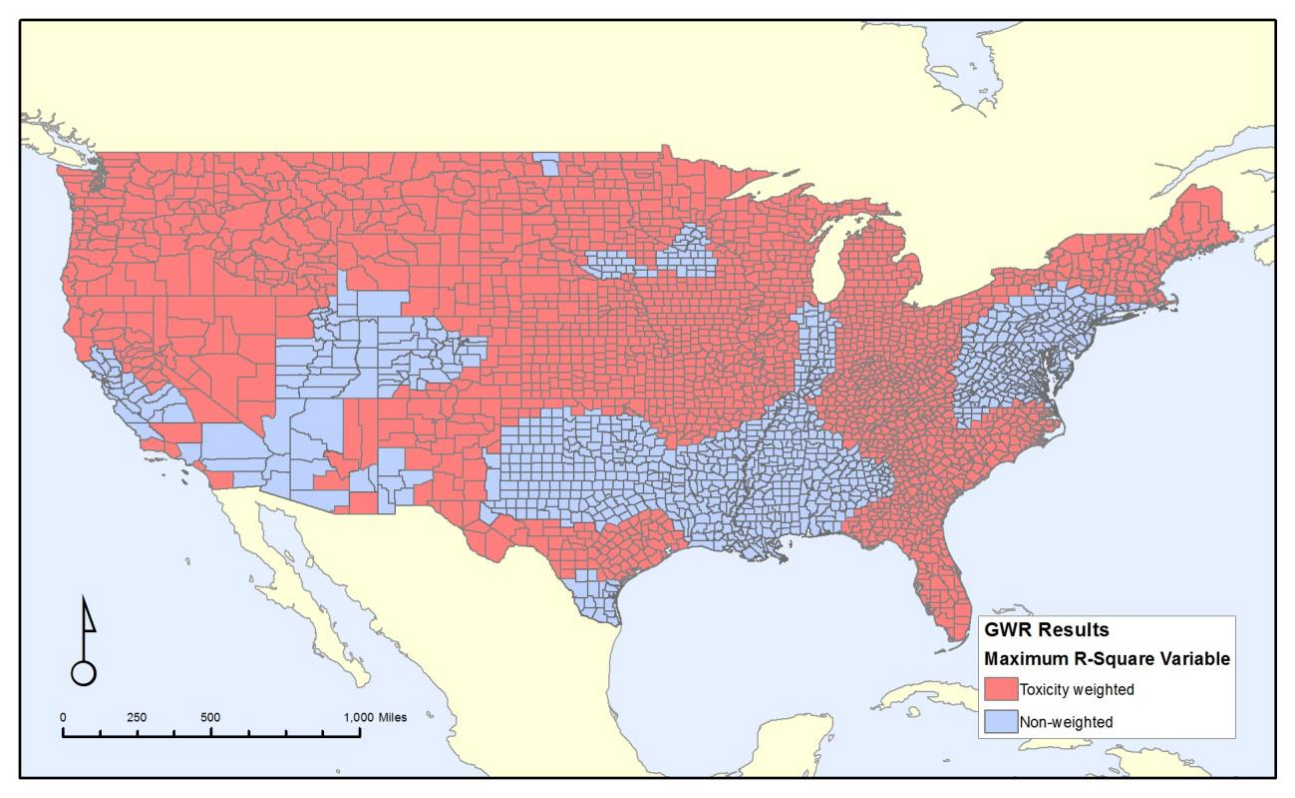
**Regions where weighting the releases by toxicity versus not weighting the releases by toxicity had the greatest local correlation with all-cancer mortality**.

Figures 4, 5 and 6 shows the attributes of the measure that led to the highest local R^2 ^value for each county. It is broken down into each of the three properties of our discharge measures: carcinogens versus non-carcinogens (Figure 4), on-site releases versus an area-weighted sum of all upstream releases (Figure 5), and whether the release amounts are weighted by toxicity values of the chemicals discharged (Figure 6). Similar to Figure 3, these maps illustrates the substantial variation from one region of the country to another, as cancer mortality in some parts of the country correlates better with the onsite variables versus the area-weighted variables. Likewise, this correlation is stronger for non-carcinogens in some regions and carcinogens in others. Thus, despite the unexpected finding from the non-spatial analyses that the non-carcinogens have a stronger correlation with cancer mortality than carcinogens, this relationship is not consistent for the entire country. There is no strong pattern throughout the country.

Figure 4 reveals two broad areas that do not conform to the national trend of non-carcinogens having a stronger relationship with cancer mortality than carcinogens. These regions, highlighted in red, are in the intermountain west and in parts of the Midwest extending to a few places along the Atlantic Coast. Figure 5 does not show a clear trend in on-site versus the area-weighted sum of upstream releases, although three areas, the Mississippi River, Florida, and an area largely east of the Appalachian Mountains extending from New York City to South Carolina, show stronger on-site release effects. For most of the United States, unsurprisingly, the toxicity-weighted measures have a stronger relationship with cancer mortality, as shown in Figure 6. However, there are some regions in the Mid-Atlantic and southern areas of the country, colored blue, where the toxicity weights do not provide a stronger relationship.

**Figure 7:**
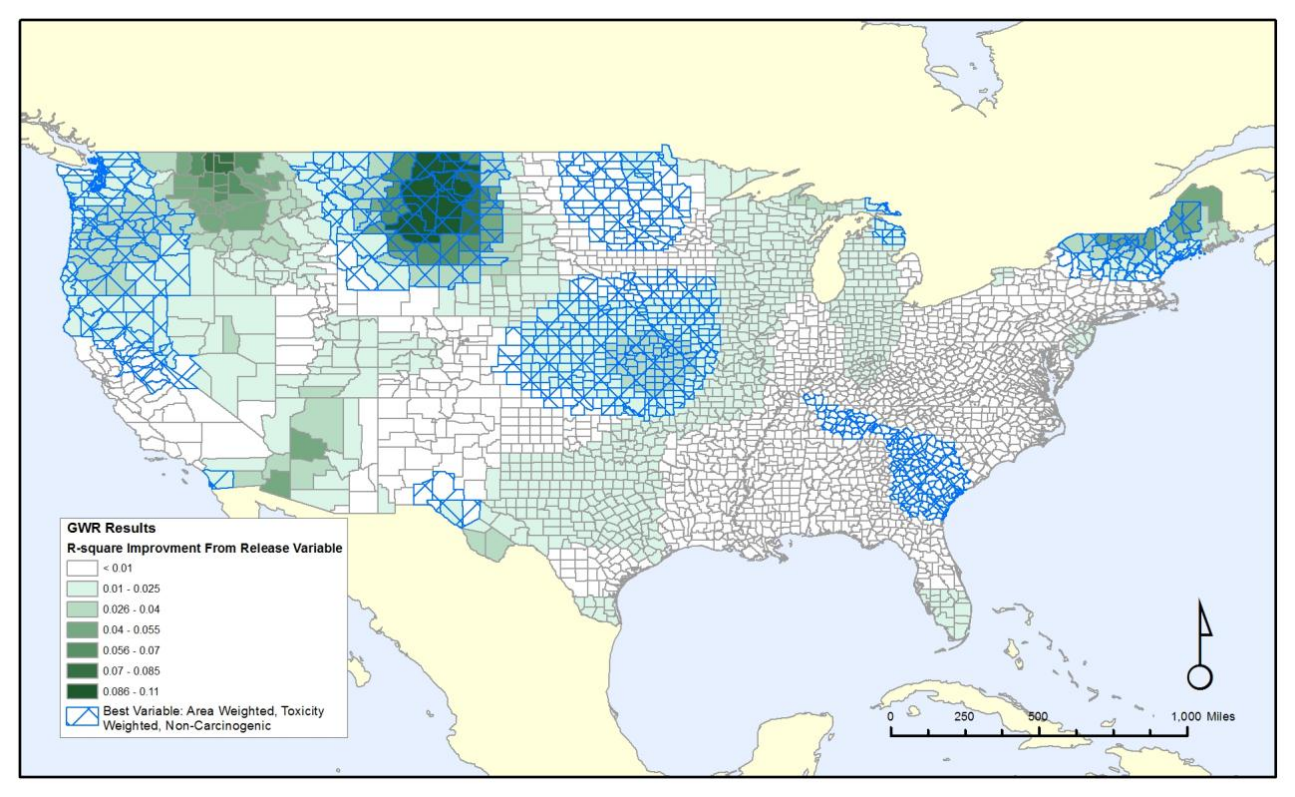
**Improvement in local R-Square by including release variable**.

Figure 7 shows the improvement in local R^2 ^over not including any release variable. This illustrates how much extra explanatory power the release variables give us compared to the demographic data and other covariates listed in Table 1. As the map shows, about half the country has very little improvement (less than 0.01 change in local R^2^), even from the best fitting release variable. Cross-hatched areas are those where the best fit was with the toxicity-weighted, area-weighted sum of non-carcinogenic releases, which is the most significant measure from the non-spatial results, and covers most regions of the country that have the greatest improvement from including pollution measures. Two large areas of substantial improvement, northern New England and the Northern Great Plains, both have the non-carcinogen releases, weighted by toxicity, as the best fit. This improvement is most dramatic in northern parts of the Great Plains, downstream from the headwaters of the Missouri and Yellowstone Rivers, which is a rural area with very little onsite releases, but with greater releases in the nearby upstream counties of Cascade and Yellowstone in Montana, which contain the cities of Great Falls and Billings respectively. Most counties in New England and all in the Northern Plains have the area-weighted measure as the best fit. Similarly, two less substantial areas of improvement in the center of the country and in the Pacific Northwest also relate to the same measure. The exceptions to this pattern are an area in the northern Rocky Mountains where the onsite toxicity-weighted release of carcinogens is highest, and an area in the southwest, centered in Arizona, where the area-weighted, non-toxicity-weighted releases of carcinogens are the strongest.

GWR analyses comparing the area-weighted non-carcinogen releases with total mortality were also conducted, but are not shown in detail to conserve space. Further information is available from the authors. The local R^2 ^values are higher than those for cancer mortality shown in Figure 3, ranging from 0.09 to 0.79, although the spatial pattern remains similar, with the highest values along the Pacific and Atlantic coasts. This greater R^2 ^value is due to the improved correlation between the covariates and the mortality rate, as the local coefficient for the pollution variable is non-significant for most of the country. Only a small area in the Great Plains and Midwest spanning from western South Dakota through Nebraska and Iowa has a significantly positive coefficient and a significantly negative coefficient is only located in the same area of West Texas that has a significantly negative coefficient in Figure 2.

## Conclusions

The results of the non-spatial analyses suggest that permitted discharges of chemical pollutants into surface waters are related to higher adjusted population mortality rates. More specifically, total non-cancer mortality is related to greater discharge quantities of chemicals classified as non-carcinogenic without need for toxicity weights or upstream discharges. For cancer mortality, the toxicity weights are necessary to detect associations between carcinogenic discharges and death rates, and for kidney disease mortality, both toxicity weights and area-weighted upstream discharges are necessary to detect discharge-mortality associations.

The cross-validation results suggest that chemicals not currently recognized as carcinogens may nevertheless play a role in contributing to cancer mortality risk. The potential carcinogenic properties of many chemicals are unknown and may be underestimated. Cross-validated results for kidney disease were significant but at a weaker level than for the non-cross-validation. There was a significant correlation between higher carcinogen releases and higher non-carcinogen releases (r = .69), so the cross-validation analysis of kidney disease may still be picking up non-carcinogen discharges. Some carcinogens such as cadmium or thallium are also recognized as causes of kidney damage [11]. In contrast, the relatively small subset of known or suspected carcinogens was related to higher cancer mortality but not higher non-cancer mortality.

Kidney and total non-cancer death rates are most strongly related to discharges in rural areas not adjacent to metropolitan areas as compared to other urban-rural settings. It is possible that downstream effects from urban to rural areas may be a contributing factor, or downstream effects from one rural area to another.

The spatial analyses illustrate the wide variation of the local R^2 ^values across the contiguous United States, as well as the variation in which model has the most explanatory power. The effects of both the chemical discharges and the covariates are not constant from one region of the country to another. Spatial models generally support the non-spatial analysis in that the releases of non-carcinogens are a better fit for the cancer mortality for most of the country (2303 out of 3109 counties) than the releases of carcinogens. For many of these counties, the improvement over not including any release variable is slight, indicating that the relative influence of chemical surface water discharges is small compared to effects of our covariates such as poverty or smoking rates. In many of the regions for which the improvement in local R^2 ^was greatest, that improvement comes from the area weighted sum of all upstream releases of non-carcinogens, adjusted for toxicity. This suggests that for some, but not all, parts of the country, upstream releases may be an important factor.

A number of hypotheses may be suggested for future research based on the findings. First, studies may undertake whether chemicals currently not recognized as carcinogens may have carcinogenic properties. The number of chemicals with established carcinogenic information, whether that information is confirmatory or not, is small relative to the number of chemicals that are manufactured or used [12] There are many chemicals used in industrial processes or that are present in drinking water for which we have no information on health risks. The results of the current study can serve to encourage future research on understanding the possible health impacts for chemicals for which there is currently limited or no information. The choice of which chemicals to investigate may be guided by those which occur at highest levels, those for which information on related chemical properties suggests a possible health concern, or those chemicals which are more prevalent in regions of the country with the strongest relationship between the total chemical discharges and cancer mortality.

Second, the effects of co-exposures or mixtures of more than one chemical deserve further investigation. Most exposure research has focused on the effects of a single agent (lead, arsenic, benzo[a]pyrene, etc.), but there is increasing recognition that exposures to multiple agents simultaneously more closely matches what people actually experience in daily life [13], and that co-exposures may have additive or synergistic effects beyond single exposures, although research on this question is limited. The exposures in the current study were not isolated as to single agents because of the large number of possible agents to investigate and because release levels of any particular agent expressed on a national scale are usually small and are often concentrated in a few regions of the country.

Based on previous research, investigations of co-exposures may best be targeted initially to combinations of single agents about which there are known effects, especially when those agents are known to have similar health impacts such as manganese and lead co-exposure impacting neurodevelopment [13], or studies that investigate mixtures of single agents that are known individually to increase cancer risk such as arsenic [14], chromium(VI) [15], PAHs [16], tetrochloroethylene [17], or others.

Third, regional variations seen in the current study are intriguing but require future investigations to attempt to understand. The northern Great Plains area highlighted in

Figure 5 is one example. This area is largely rural and sparsely populated. It may be that rural areas, at least in some circumstances, are less impacted by environmental contaminants than urban areas, such that, when an environmental pollutant source (such as PCS discharges) is present in a rural area, that source represents a unique "spike" in exposures relative to background, whereas in urban areas with the same PCS pollutant source, the additional contribution of this source to health outcomes may be harder to detect against a background of other pollutants from industry or transportation.

Fourth, spatial variation in the contributions of area-weighted and on-site discharges suggests that area-weighted or upstream discharges may be important for some areas, whereas local discharges are more important for others (Figure 5). It is difficult to identify a pattern that can account for this variation; on-site discharges are relatively more important along the entire Mississippi River, but other major river systems don't show this pattern. Some major population centers are in areas where on-site discharges are more important, but other population centers are in areas where area-weighted scores had stronger effects. Regional variation in the composition of chemicals discharged may play a role in this spatial variation, as some chemicals or combinations of interacting chemicals may be present in one area but not in others. Regions to examine for these effects include the Northern Rockies and Arizona, where the measure of carcinogen releases instead of the non-carcinogen releases added substantial explanatory ability to the model, as well as areas in the Northern Plains and New England, which showed the strongest relationship between non-carcinogenic releases and cancer mortality. Similarly, there may be regional variation in how far downstream chemicals travel from the discharge site. Both properties of the chemical, such as its molecular weight, and properties of the stream, such as how fast it is flowing, could affect the distance the chemical travels. Accounting for molecular weight of airborne pollutants can improve models of atmospheric releases and public health outcomes [18], and a similar strategy may be useful when examining water-borne discharges.

Limits of the study include the ecological design, the selection of a partial list of chemicals with ingestion toxicity weights, the knowledge that the health impacts of mixtures are poorly understood, and the imperfect time relationships between discharges and mortality. Kidney disease was selected as one diagnostic sub-group for study but others, such as bladder cancer [19] could also have been investigated. We do not account for additional environmental variables that may be related to cancer or non-cancer risks, including geographic variation in levels of UV-B [20,21], nitrates from non-point pollution agricultural sources [22], or traffic emissions. The results of the study must be taken as exploratory, but do show possible connections between greater permitted discharges of toxic chemicals into surface water and human health consequences, with potentially important geographic variations in the impacts of these discharges and in the particular discharges and health outcomes of greatest concern.

## Methods

### Design

The study employs a county-level, ecological secondary data analysis. Dependent variables are population age-adjusted mortality rates (e.g., cancer mortality rates), and are statistically associated with independent variables (e.g., releases of carcinogens into surface waters) in the context of controlling for covariates (e.g., race/ethnicity, poverty rates, physician supply). Variables are described in further detail below.

The design also includes comparative findings for rural and urban areas. Counties were classified using the US Department of Agriculture's urban-influence codes (UICs) to identify metropolitan areas (codes 1 and 2), non-metropolitan areas adjacent to metropolitan areas (codes 3,4,5,6,7,9 and 10), and non-metropolitan areas not adjacent to metropolitan areas (codes 8,11 and12).

### Data sources and variables

The EPA's Discharge Monitoring Report (DMR) database, which includes data from the Permit Compliance System (PCS) and the Integrated Compliance Information System - National Pollutant Discharge Elimination System (ICIS-NPDES), was used to measure the location, type, and quantity of water pollution discharges [23]. The DMR database provides information on companies that have been issued permits to discharge wastewater into rivers or streams, including data on the amounts and types of chemicals discharged. An exported Oracle database was provided to us by the EPA containing the DMR data for the year 2007. The pollutant loading table in the database included 322,113 records of aggregate discharge measurements from 30,228 unique facilities. One thousand one hundred nine (1,109) parameters are included in the data, from basic water chemistry information (pH, temperature, etc) to concentrations of various compounds classified as "pollutants" by the EPA (n = 729). Not all records contain values for all parameters; each record contains values for one parameter, relevant to that facilities' permit. Of the pollutants, a total of 518 unique Chemical Abstract Service (CAS) registry numbers were identified in the data. Of those 518 CAS registry numbers, we initially limited the analysis to discharges of 73 chemicals selected based on their possible human health impacts. We chose a subset of chemicals rather than attempting to use all chemicals because of the extensive time demands required to find, clean, and aggregate chemical-specific discharge data across the 322,113 discharge records in the DMR data. Selecting only those records containing a chemical of interest left us with 55,183 records. We also limited the data points used in the analysis by removing all records with a release value across all chemicals of interest of zero (n = 20,948). Next, we removed all records that fell outside of the contiguous United States (n = 13,197), and all records whose latitude/longitude coordinate fields contained values of "0" or other anomalous values (n = 143). Finally, we removed all records wherein a single facility listed the same discharge value for all releases as this was clearly reported in error (n = 56). Once these edits were completed, we were left with a database of 19,824 permitted discharges from 10,395 individual facilities which were used in development of subsequent analyses.

To aggregate discharges from upstream sources into downstream geographic areas, we utilized the Watershed Boundary Dataset, a multi-level spatial dataset for watersheds created and maintained by the Natural Resource Conservation Service (NRCS) and published as part of the National Hydrography Dataset (NHD) [24]. The data were downloaded from the NHD server as a single file for the United States. We extracted the Sixth level (12 digit) watershed and checked the relevant upstream and downstream fields within the database to ensure that we could connect the upstream to downstream flows. Finally, we summed the discharges per chemical within each watershed for use in later analysis and aggregation.

### Toxicity weighted and un-weighted discharges

Chemicals vary in their toxicity, such that a given amount of exposure may be harmless for one chemical and deadly for another. Efforts have been undertaken to estimate toxicity weights for specific chemicals [25]; currently there are weights available for some but not all chemicals included in the DMR database. From our initial list we selected all 30 non-carcinogenic and all 8 carcinogenic chemicals with ingestion toxicity weights as established by the EPA [26]. Carcinogens were included if they were categorized as class 1, 2a or 2b by the International Agency for Research on Cancer (IARC) or as a Known or Probable carcinogen by the National Toxicology Program (NTP). For consistency, analyses and reports presented in this paper for toxicity weighted and non-weighted findings use the same subset of 38 chemicals. The final list of chemicals with weights is presented in Table 5. Although some of the listed non-carcinogens have carcinogenic properties (e.g., cadmium) we included only chemicals with established toxicity weights for ingestion exposures, not inhalation exposures.

**Table 5:** List of chemicals used in analyses

	Chemical Name	Toxicity Weight
Non-carcinogens	2,4-Dinitrophenol	500

	1,1,1-Trichloroethane	0.5

	Methoxychlor	200

	1,1-Dichloroethylene	20

	Hexachlorocyclopentadiene	170

	Dinoseb	1000

	2,4-D	200

	o-Dichlorobenzene	11

	1,2-Dibromo-3-chloropropane	5000

	Styrene	5

	Toluene	13

	Chlorobenzene	50

	Phenol	3.3

	1,2,4-Trichlorobenzene	100

	Xylene	5

	Carbofuran	1200

	Atrazine	56

	Lead	18000

	Manganese	7.1

	Mercury	10000

	Nickel	20

	Thallium	14000

	Antimony	2500

	Barium	5

	Beryllium	500

	Cadmium	2000

	Chromium	330

	Copper	1500

	Selenium	200

	Chlorine	10

Carcinogens	Lindane	110000

	Benzene	55000

	1,1,2-Trichloroethane	5700

	Ethylbenzene	1100

	p-Dichlorobenzene	2400

	Di(2-ethylhexyl) phthalate	14000

	Polychlorinated biphenyls	2000000

	Arsenic	1500000

For toxicity weighted analyses, the values for each chemical were multiplied by the weight for that chemical. Toxicity weighted and unweighted quantities for each county were then summed across all carcinogens, and again across all non-carcinogen chemicals. Amounts of these summed chemical discharges were not normally distributed across counties, so we calculated the natural log of discharge amounts for analysis. All discharges are expressed as the log of kg per year.

### Onsite and area-weighted upstream discharges

Onsite discharges were measured as the simple sum of the log carcinogen and non-carcinogen chemical discharges present in each county. These sums were computed for both toxicity weighted and non-weighted discharges. Discharges into waterways can flow downstream to impact communities where there may be few or even no on-site releases. To account for the impact of upstream discharges, we develop a measure that allows discharges to accumulate throughout a river system. We also want to account for the likelihood that releases upstream from a location will have a smaller impact on that location than nearby releases. We perform this accounting by using a weighted sum of all upstream releases, dividing each release by the area of the watersheds between the release site and the impact site. The following equation gives how this is calculated.

$$\pi_{s}=\underset{w\geq s}{\sum }\frac{\rho_{w}}{area_{w\to s}}$$

Here, *π_s _*is the pollution score for the watershed, *ρ_w _*is the summed releases for that watershed, *w *≥ *s *denotes all watersheds upstream of shed *s*, including shed *s *itself, and *area_w→S _*denotes the area in acres of all watersheds between sheds *w *and *s*, including both *w *and *s*. When *w *= *s*, this reduces to the area of that watershed. We employ this reduction to account for the likelihood that releases far upstream of a location will have less influence on that location than nearby releases.

### Population-weighted county-level discharges for both onsite and areas-weighted upstream discharges

Because the demographic and mortality variables are reported for each county, while the discharge variables are calculated for each watershed, we transformed the release variables from the smaller watersheds to county-level summaries to conduct statistical analysis at the county level. A simple summation of the releases within the counties is insufficient because of potential discrepancies within each county between where the residents live and where the releases take place. As an extreme example, imagine a county split between two watersheds; the first watershed has all the releases but none of the population, while the second watershed has all the population but none of the releases. Even though there are chemical discharges to streams within the county, none of the population is exposed to those releases. Therefore, we estimated the population living within each watershed and county intersection. We used the LandScan Global dataset [27] which estimates population at a grid with cells approximately 1 km by 1 km in size. We then created a population-weighted average exposure in each county by applying the following formula:

$$e_{c}=\frac{\sum_{s\cap c}\pi_{s}*pop_{s,c}}{\sum_{s\cap c}pop_{s,c}}$$

Here, *s *is a watershed, *c *is the county, *pop_s, c _*is the estimated population in the watershed/county intersection, *π_s _*is the pollution score for the watershed, and *e_c _*is the total exposure score for the county. The denominator of the fraction is simply the population of the county, but is shown as the sum of the population of all watershed/county intersections to illustrate the weighted average nature of the calculation. We calculated values for both onsite and area-weighted exposures. The onsite calculation replaces *π_s _*with the release variable *ρ_s_*.

### Outcome measures

Health outcome data were drawn from the public CDC mortality files for the years 2003-2007. We selected a five-year aggregate period to acquire more stable estimates than would be possible by selecting only one year, and choose the most recent five-year period available from the CDC at the time of the study, recognizing that this creates an imperfect match between the mortality observation period and the chemical discharge period. We are forced to assume that PCS discharge quantities at the county level are stable over time, such that later discharges provide a reasonable estimate of earlier discharges.

From the CDC we found the annual age-adjusted mortality rates per 100,000 for 1) all cancer (ICD-10 codes C00-C97 malignant neoplasms); 2) chronic or unspecified non-cancer kidney disease (ICD-10 diagnostic GR113 codes 99, 100 and 101; the uncommon code 98 reflecting 'acute and rapidly progressive' disease was excluded); and 3) all non-cancer mortality causes causes combined, excluding accidents, suicide and homicide. Kidney disease was selected as one category because of previous research suggesting that kidney disease may be particularly sensitive to exposure to water pollutants, especially heavy metals [28-31]. Rates were age-adjusted using the standard 2000 US Census population.

### Covariates

Other variables were measured from the 2007 Area Resource File and CDC 2006 Behavioral Risk Factor Surveillance System (BRFSS) survey data. Covariates include county-level measures of adult smoking rates, college education rates, poverty rates, race/ethnicity percentages, physician per capita supply, and adult obesity rates.

### Analysis

Data analyses included calculation of descriptive statistics and examination for multicollinearity, followed by non-spatial and spatial analyses. For the non-spatial analyses, we examined associations between chemical discharges and mortality through a series of linear multiple regression models designed to build on one another to test whether refinements to the specification of the discharge variables improved their capacity to account for mortality rates. Specifically, we ran a series of five sets of analyses, and within each set we examined the three primary outcomes of interest including cancer mortality rates, total non-cancer mortality rates, and kidney disease mortality rates. In Sets 1 through 3 below, carcinogen discharges were used in models of cancer mortality and non-carcinogen discharges were used in models of non-cancer mortality. In Set 4, models were cross-validated by using carcinogen discharges in non-cancer mortality models, and by using non-carcinogen discharges in cancer mortality models. The five sets in sequence were:

1. Onsite discharges not toxicity weighted

2. Onsite discharges with toxicity weights

3. Area weighted upstream discharges with toxicity weights

4. Area weighted upstream discharges with toxicity weights cross-validated.

5. Area weighted upstream discharges with toxicity weights separately for metropolitan, non-metropolitan adjacent, and non-metropolitan non-adjacent counties

Spatial analyses included a series of seven geographically weighted regressions (GWR) [10,32]. This approach recognizes that the relationships between the independent and dependent variables in a standard regression analysis may mask spatial variation in the relationships, such that the relationship may be strong in one part of the study area yet weak in another part. This could arise in our study because we are aggregating the releases of many chemicals together, and spatial variation in the composition of the chemical discharges could result in spatial variation in the relationship between discharges and public health outcomes. The GWR procedure cycles through each county and conducts a multiple linear regression for each county in the dataset, using only the nearby counties. In this study, we used the 30 nearest counties. This approach provides a local R^2 ^value and local coefficients for each county based on its thirty nearest neighbors, rather than simply reporting a single result for the entire dataset. Each of them compared cancer mortality with the same demographic covariates as in the non-spatial regressions, and one of the following pollutant discharge variables.

A. Area weighted carcinogen releases, toxicity weighted

B. Area weighted carcinogen releases, not toxicity weighted

C. Area weighted non-carcinogen releases, toxicity weighted

D. Onsite carcinogen releases, toxicity weighted

E. Onsite carcinogen releases not toxicity weighted

F. Onsite non-carcinogen releases, toxicity weighted

G. Onsite non-carcinogen releases not toxicity weighted

The eighth possible analysis, using the area weighted non-carcinogen releases, not toxicity weighted, was not completed because the GWR failed to evaluate because of local multicollinearity errors, even when the number of neighbors was increased to 300 counties. We did not examine the geographic patterns of kidney mortality because the suppression of some counties' data due to small numbers of cases precluded spatial analysis. We decided to limit the spatial analysis to cancer mortality to conserve space, but results for non-cancer mortality are briefly described in text in the Results section.

## Competing interests

The authors declare that they have no competing interests.

## Authors' contributions

MH conceived the study and contributed to the design, analysis, interpretation of results, and writing the manuscript. JC led the spatial analysis and contributed to interpretation of results and writing. EF contributed to the spatial analysis, interpretation of results and writing. JL contributed to the statistical analysis, interpretation of results and writing. MA contributed to database creation, study design, and writing. All authors read and approved the final manuscript.

## Note

Support for this study was provided by the Office of Rural Health Policy, Health Resources and Services Administration, PHS Grant No. 1 U1CRH10664-01-00.
